# Goblet cell metaplasia and mucin alterations in biliary epithelial cells during *Opisthorchis viverrini* infection in rodent models: Insights into host susceptibility and defense mechanisms

**DOI:** 10.14202/vetworld.2025.534-546

**Published:** 2025-03-09

**Authors:** Woro Danur Wendo, Theerayut Thongrin, Prasarn Tangkawattana, Peerapol Sukon, Sutas Suttiprapa, Prasert Saichua, Watcharapol Suyapoh, Sirikachorn Tangkawattana

**Affiliations:** 1 Graduate School, Faculty of Veterinary Medicine, Khon Kaen University, Khon Kaen, 40002, Thailand; 2 Department of Anatomy, Faculty of Veterinary Medicine, Khon Kaen University, Khon Kaen, 40002, Thailand; 3 Department of Tropical Medicine, Faculty of Medicine, Khon Kaen University, Khon Kaen, 40002, Thailand; 4 Department of Veterinary Science, Faculty of Veterinary Science, Prince of Songkla University, Songkhla, 90110, Thailand; 5 Department of Pathobiology, Faculty of Veterinary Medicine, Khon Kaen University, Khon Kaen, 40002, Thailand

**Keywords:** Bile duct proliferation, goblet cell metaplasia, histopathology, host susceptibility, mucin dynamics, *Opisthorchis viverrini*, rodents

## Abstract

**Background and Aim:**

Chronic *Opisthorchis viverrini* (OV) infection induces significant biliary changes and is a major risk factor for cholangiocarcinoma. However, the role of goblet cell metaplasia (GCM) and mucin dynamics in host defense and parasite persistence remains poorly understood. This study aims to characterize biliary histological changes, particularly mucin types, and compare responses between susceptible (hamsters) and non-susceptible (mice) hosts during early to chronic OV infection.

**Materials and Methods:**

Thirty-five male golden Syrian hamsters and 35 male BALB/c mice were divided into infected and control groups. Infected animals received 50 OV metacercariae through gastric intubation and were sacrificed on days 1, 2, 7, 14, 28, and 56 post-infection. Histological, histochemical (Alcian Blue, periodic Acid-Schiff, and high iron diamine), and immunohistochemical (Bromodeoxyuridine [BrdU]) analyses were performed to assess mucin production, GCM, and bile duct proliferation.

**Results:**

Mice demonstrated an early, robust biliary response with pronounced hyperplasia and GCM characterized by acid mucin overproduction during the acute phase (days 1–28). Conversely, hamsters exhibited delayed biliary proliferation and GCM, with predominant sulfated mucins appearing during the chronic phase (days 28–56). BrdU immunoreactivity indicated earlier and stronger bile duct epithelial proliferation in mice, correlating with worm clearance by day 28. In hamsters, mucosal changes supported worm survival, as evidenced by continued parasite presence and egg production. Statistical analyses confirmed significant differences in mucin types and hyperplasia between species across infection stages.

**Conclusion:**

Distinct mucosal responses in hamsters and mice reflect their susceptibility to OV infection. Acid mucins in mice facilitate worm expulsion, while sulfated mucins in hamsters appear to promote parasite persistence. These findings highlight the dual roles of mucins in host defense and parasite survival, providing insight into mechanisms underlying susceptibility and resistance in OV infections.

## INTRODUCTION

Chronic infection with the liver fluke *Opisthorchis viverrini* (OV) causes significant hepatobiliary damage in humans, including cholangitis (bile duct inflammation), periductal fibrosis, and eventual progression to cholangiocarcinoma (CCA), a fatal cancer prevalent in Southeast Asia [1–3]. Experimental studies using a hamster model demonstrated the establishment of OV infection, worm survival, and high pathogenicity of OV infection and clarified the pathogenesis of CCA (cholangiocarcinogenesis) [1, 4, 5]. Notably, <25% of infections progress to advanced stages [6], and CCA occurs in <1% of patients infected with OV [7], indicating variability in pathogenicity among hosts. In addition, Srisai *et al*. [8] and Wonkchalee *et al*. [9] demonstrated the comparative susceptibility of laboratory rodents to OV infection. This suggests that hamsters are susceptible to OV infection, whereas mice are considered non-susceptible [10]. The distinct host-parasite interactions may be key to combating parasites. The infection process begins when metacercariae are ingested, migrate to the duodenum within a few hours, and move to the biliary system within 24 h [11]. OV establishment, survival, and pathogenicity are closely linked to histopathological changes in the hepatobiliary system, including bile duct proliferation, inflammation (cholangitis), and goblet cell metaplasia (GCM) [12, 13].

Worms obtain nutrients from the host niche, where the mucosal surface is covered with glycoprotein mucin produced by goblet cells. In response, the host increases the number of goblet cells and modified mucins. Goblet cell hyperplasia and shifts toward more sialylated or sulfated mucins are key host defense mechanisms aimed at parasite expulsion [14–16]. This response, crucial for worm expulsion, has been observed in rodent models, especially in non-susceptible hosts [17–19]. However, adaptations such as GCM may also facilitate OV persistence and pathogenicity [12]. Although mucin dynamics have been well studied in gastrointestinal helminth infections, their regulation and functional roles in biliary infections remain poorly understood. Mucins are characterized by specific glycan types at their terminal structures, which significantly influence their bioactivity [20–22]. Histochemical studies have revealed that mucin types vary among species and play critical roles in the survival strategies of helminths [23, 24]. In response to OV infection, the transformation of bile duct epithelial cells into a mucin-producing gland-like structure or GCM is critical for understanding infection persistence and pathology [25–27]. The balance between mucin-mediated defense and its exploitation by the parasite requires further investigation.

This study aimed to characterize changes in mucin expression patterns, highlighting GCM, and to compare mucin profiles between susceptible and non-susceptible hosts at different infection stages. The study will explore host-specific mucin modifications in the bile duct to influence parasite survival and disease progression.

## MATERIALS AND METHODS

### Ethical approval

This study was approved by the Animal Ethics Committee, Khon Kaen University (approval numbers IACUC-KKU-57/63 and 94/66).

### Study period and location

The study was conducted from October 2021 to December 2023 at the Faculty of Medicine, Faculty of Veterinary Medicine, Khon Kaen University, Thailand.

### Animal models

Based on the animal model of opisthorchiasis [12], this study used 35 male golden Syrian hamsters (*Mesocricetus auratus*) aged 6 weeks and 35 males from a non-susceptible animal model, BALB/c mice aged 6 weeks, a non-susceptible model. The hamsters were housed under conventional conditions at the Animal Model Facility, Faculty of Medicine, Khon Kaen University, while the mice were housed in Biosafety Level 3 conditions at the Northeast Animal Laboratory Center, Khon Kaen University. All animals had access to a stock diet and water *ad libitum*, in accordance with National Experimental Animal Center Standards. The animals of each model were divided into six subgroups based on day post-infection (p.i.) and had a non-infection group (5 animals) as a negative control.

### OV: Parasite preparation and animal infection

Infected larvae were collected from the final pellet of digested OV-infected cyprinoids from natural water sources in Northeast Thailand. Fish were digested using HCl and pepsin solutions, followed by filtration through a 350 μm pore size mesh. Metacercariae were identified under a stereomicroscope as previously described by Kaewkes [28]. For infection, each animal in the infected group was administered 50 OV metacercariae through intragastric intubation. Before euthanasia, all hamsters and mice were intraperitoneally injected with the cell proliferation marker bromodeoxyuridine (BrdU, Sigma-Aldrich^®^, St. Louis, MO, USA, B5002) at a dose of 40 mg/kg. Animals were euthanized by isoflurane inhalation on days 1, 2, 7, 14, 28, and 56, following the American Veterinary Medical Association guidelines (2013) (https://www.in.gov/boah/files/AVMA_Euthanasia_Guidelines.pdf). Worm recovery was assessed by examining stool samples from eggs using the Formalin-ethyl acetate concentration technique, which is the gold standard method [29]. Eggs were identified morphologically under a light microscope based on shell surface features, knobs, operculum, and shape [30].

### Sample collection and histopathological examination

All liver lobes, including the biliary apparatus, were examined for gross lesions and then fixed individually in 10% neutral buffered formalin. After 24 h, dissected tissues were embedded in paraffin blocks, following routine histological procedures. Sections in 4 μm-thickness were prepared on coated slides for histopathological, histochemical, and immunohistochemical analyses. Deparaffinized and hydrated tissues were stained with hematoxylin-eosin for histopathological identification, as described by Thongrin *et al*. [10].

### Histochemical analysis

#### Neutral and acid mucin detection using alcian blue pH2.5-periodic acid Schiff (AB-PAS)

AB stains sulfated and carboxylated acid mucins blue, whereas PAS stains neutral mucins with magenta oxidizing vicinal diols. The AB pH 2.5-PAS combination distinguishes between acid (mucus) and neutral (serous) mucins or their mixtures, which appear purple. Staining was performed with minor modifications to the protocol described by Bancroft and Gamble [31].

#### Acid-sulfated mucin detection using high iron diamine (HID)-AB-PAS

HID histochemical staining is used to detect acid mucins containing sulfate esters [31]. The HID-AB-PAS combination distinguished sulfated acid, sialylated acid, and neutral mucins, which appear black-brownish, turquoise-blue, and magenta, respectively. Hydrated slides were incubated in HID reagent for 20 h a room temperature (25°C), followed by AB-PAS staining with minor modifications to the protocol reported by Ueda *et al*. [32].

#### Immunodetection of the cell proliferation marker, BrdU

Newly synthesized DNA in actively proliferating cells was detected using anti-BrdU–specific antibodies. Biliary epithelial cells were incubated with sheep polyclonal anti-(BrdU, Abcam plc, Cambridge, UK, ab1893) diluted 1:500 in phosphate-buffered saline azide using the Avidin–biotin immunoperoxidase technique [12]. Biotinylated rabbit anti-sheep immunoglobulin G (1:200, Abcam plc, Cambridge, UK, ab97130) was used as the secondary antibody. Immunoreactivity was visualized using 0.05% 3,3′-Diaminobenzidine-tetrahydrochloride (Sigma Aldrich, Germany) in 0.003% H_2_O_2_. Mayer’s hematoxylin was used as a counterstain, followed by dehydration, clearing, and mounting with Permount (USA). Proliferative and non-labeled tissues were included as positive and negative controls, respectively.

### Statistical analysis

Histopathological grading was evaluated semi-quantitatively based on the criteria presented in Table 1 [12]. Biliary epithelial mucin production and bile duct epithelial cell proliferation were assessed using the mucin index and BrdU index, respectively [33, 34]. The statistical analysis of histopathological and biochemical features was conducted to assess differences in biliary epithelial responses between hamsters and mice across various stages of *O. viverrini* infection. Normality of the data was evaluated using the Shapiro–Wilk test, and appropriate parametric or non-parametric tests were applied based on the results. For normally distributed data, comparisons between groups were performed using independent t-tests (for two groups) or one-way analysis of variance (ANOVA) (for multiple groups), followed by *post hoc* Tukey’s Honestly Significant Difference tests for pairwise comparisons. Non-normally distributed data were analyzed using Mann–Whitney U tests (for two groups) or Kruskal–Wallis tests (for multiple groups), with *post hoc* Dunn’s tests to identify significant pairwise differences [35]. To evaluate changes in parameters over time within each group, repeated measures ANOVA was applied for normally distributed data, while the Friedman test was used for non-parametric repeated measures. *Post hoc* adjustments, including the Greenhouse–Geisser correction, were implemented when sphericity assumptions were violated. Correlations between histopathological features, such as GCM, mucin production, and the BrdU index, were assessed using Pearson correlation for normally distributed data and Spearman correlation for non-linear or non-normal data. Mixed-effects models were employed to account for repeated measures within subjects and to evaluate the influence of covariates, such as infection duration and host species, on histological and mucin production outcomes. Effect sizes were reported alongside p-values to quantify the magnitude of observed differences or associations. Statistical significance was set at p < 0.05 for all analyses. Data visualization, including line graphs and boxplots, was used to illustrate trends and group differences effectively. All analyses were performed using the Statistical Package for the Social Sciences version 28 (IBM Corp., NY, USA) to ensure precision and reproducibility.

**Table 1:** Semi-quantitative score criteria and quantitative formula of the biliary histopathology.

Parameter	Score criteria and formula	References
Inflammation	0 ≤ 1%	[12]
	1=1–<25%	
	2=25–50%	
	3 ≥ 50%	
Hyperplasia	Lesion structures in the total portal triad population	[12]
	0 ≤1%	
	1=1–<25%	
	2=25–50%	
	3 ≥ 50%	
Goblet cell metaplasia	Lesion structures in the total portal triad population	[12]
	0 ≤ 1%	
	1=1–<25%	
	2=25–50%	
	3 ≥ 50%	
Proliferation (quantitative): BrdU index	Number of BrdU-immunoreactive biliary epithelial cells/total biliary epithelial cells × 100%	[12]
Mucin production (quantitative): Mucin index	Area of mucin/total area biliary epithelial × 100%	[34]

BrdU=Bromodeoxyuridine

## RESULTS

### Infection status and worm recovery

Both animal models developed opisthorchiasis with generally no clinical signs that were specifically distinguished from normal non-infected animals during early infection (data not shown). The animals in the infection group were fed 50 metacercariae through an intragastric tube. The fluke grew up as a newly excysted juvenile worm and developed in the biliary apparatus. Worm recovery was monitored using microscopy by examining eggs from feces and the presence of worms in the biliary tissues. There were differences in general features, maturity, and distribution within periods of infection between the two animal models. Juvenile OVs entered the bile ducts of hamsters and mice on day 1 p.i., yet, adult OVs reached full maturation only in hamsters. The complete structures of gonad and egg fecundity were observed on day 28. These findings, along with the eggs, were detected in the hamster stool samples (Table 2). Adult OVs were also distributed at the common bile duct and gallbladder, and they were occasionally seen in the pancreatic duct of hamsters but not in the mice extrahepatic biliary system. In contrast, OVs only existed in the intrahepatic bile ducts of mice up to 28 p.i. without complete maturation. As a result, they failed to produce eggs and were not detected in the stool. OV was assumed to be completely expelled after 28 p.i. due to the absence of the worm in the biliary apparatus (Table 2).

**Table 2:** Qualitative worm distribution and egg recovery under microscopic observation.

Day after infection	OV distribution		Eggs detection		OV and egg detection	
		
	Infected group		Infected group		Non-infected group	
		
	Hamsters	Mice	Hamsters	Mice	Hamsters	Mice
1	+	+	-	-	-	-
2	+	+	-	-	-	-
7	+	+	-	-	-	-
14	+	+	-	-	-	-
28	+	+	+	-	-	-
56	+	-	+	-	-	-

H=Hamster, M=Mice, +=detected, =Absent, OV=*Opisthorchis viverrini*

### Histopathological lesions

OV infection affects the liver and biliary system over time. Histologically, no inflammatory cells or biliary changes were observed in the biliary apparatus tissues of non-infected hamsters and mice. Alterations in the biliary apparatus due to OV infection were evident from the early phase, contrasting with non-infected tissues, which showed no observable histological changes. The progression from acute to chronic phase was associated with various lesions, which were examined using routine histopathological staining, immunohistochemistry, and histochemical techniques. In the acute phase, host responses were characterized by the infiltration of small amounts of polymorphonuclear cells predominated by eosinophils, followed by the recruitment of mononuclear cells, such as lymphocytes, plasma cells, and macrophages, during the chronic phase. In addition, bile duct epithelial cells underwent proliferation and transformation, with the development of hyperplastic lesions and GCM. Microscopically, the hyperplastic biliary epithelium was surrounded by inflammatory cells, exhibiting changes from mild to severe, along with periductal fibrosis. These lesions were particularly notable in the first-order bile ducts. Hamsters displayed a more severe inflammatory response than mice in both the intrahepatic and extrahepatic bile ducts, particularly in the late phase, whereas mice exhibited more progressive proliferation, bile duct hyperplasia, and GCM during the acute phase. From the beginning, inflammation in the hamster model increased slower than that in the mice but remained stronger in the chronic phase, whereas mice showed a diminished response in the later stages of infection. Periductal fibrosis was established in the late phase, indicating chronic inflammation, with hamsters exhibiting greater progression than mice.

### Hyperplasia

**Figure 1:**
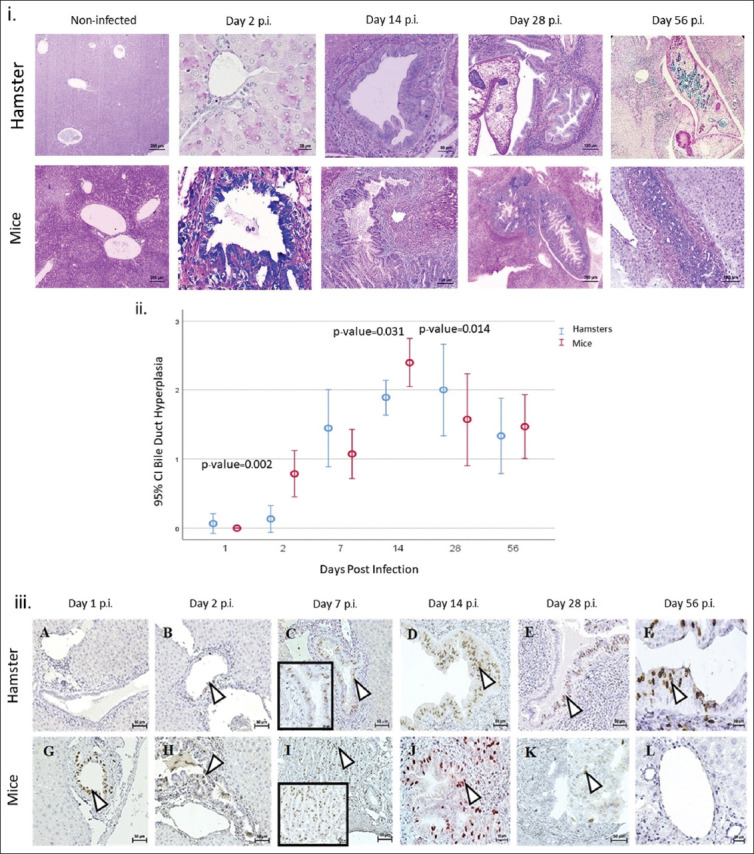
Histopathologic study of intrahepatic bile duct hyperplasia in hamsters and mice. (i) Histopathologic features of hyperplasia in the intrahepatic bile ducts of hamsters (left column, B-F) and mice (right column, H-L) following *Opisthorchis viverrini* (OV) infection. Hyperplastic lesions in the biliary epithelium (arrows) are observed on days 2 (B, H), 7 (C, I), 14 (D, J), 28 (E, K), and 56 (F, L). Aggressive hyperplasia (E, F) is noted at the site of host–OV contact (*). Normal non-infected hamster (A, arrowheads) and mouse (G, arrowheads) bile ducts show no lumen enlargement or epithelial cell hyperplasia. (ii) Graph illustrating the trend in hyperplasia lesion scores in OV-infected hamsters and mice on days 1, 2, 7, 14, 28, and 56 post-infection in the intrahepatic bile duct. Significant differences between animals are indicated by p < 0.05 (*). (iii) BrdU immunohistochemistry of biliary cells in hamsters (A-F) and mice (G-L) indicates proliferation. BrdU=Bromodeoxyuridine.

Hyperplastic epithelial cells were observed in OV-infected hamsters and mice, particularly in large first-order bile ducts. Normal bile ducts showed no lesions, indicating activation of cholangiocytes by OV antigens or excretory/secretory products. Hyperplastic lesions were characterized by stratified or folded epithelial cells, with some forming papillary hyperplasia (Figure 1i). Despite the presence of lesions, maximum hyperplasia scores were not reached, reflecting localized rather than widespread changes.

Mice exhibited earlier and more extensive hyperplasia than hamsters, with significant differences on day 2 and day 14 p.i. (p = 0.002 and p = 0.031, respectively) before declining. From day 28 p.i., hyperplasia in mice decreased the score, whereas hamsters showed advanced lesions with hyperplasia and inflammation (Figures 1i and 1ii). The mucosal changes were initially identified as papillary hyperplasia and developed into long papillary formation, highlighting a histopathological distinction from what was observed in hamsters.

### BrdU immunoreactivity and bile duct epithelial cell proliferation

BrdU immunoreactivity (Figure 1iii) confirmed the mitotic activity of bile duct epithelial cells during the S (synthesis) phase. This marker was detected only in OV-infected cholangiocytes, and no reactivity was observed in non-infected tissues. In mice, intense BrdU labeling was observed in the bile duct from day 1 p.i. (Figure 1iii H, arrow), whereas no reactivity was found in hamster cholangiocytes at the same time point (Figure 1iii B). Both models showed abundant immunoreactivity by day 7 and day 14 p.i. (Figure 1iii C, D, I, J). However, in the later stages (Figure 1iii E, F, K, L), contrasting trends indicated strong biliary epithelial proliferation induced by OV infection. Figures 1iii and Table 3 illustrate the BrdU index trends in the first- and second-order bile ducts from day 1 to 56 p.i. The BrdU index, which is the ratio of BrdU-labeled to total biliary epithelial cells, indicates earlier and more aggressive cholangiocyte proliferation in mice than in hamsters. After reaching their peak indices, mice and hamsters exhibit opposite trends in first-order bile ducts but similar patterns in second-order ducts.

**Table 3:** Statistical correlation among histopathological lesions in the intrahepatic bile duct of hamsters and mice indicated by Spearman’s coefficient correlation.

Comparison	Hamsters		Mice	
	
	Spearman’s coefficient correlation (r_s_)	p-value	Spearman’s coefficient correlation (r_s_)	p-value
Hyperplasia versus				
Proliferation (BrdU index)	0.825^+++^	0.000**	0.027	0.802
Goblet cell metaplasia	0.330	0.008**	0.799^++^	0.000**
Goblet cell metaplasia mixed mucin	0.330	0.008**	0.383	0.000**
Goblet cell metaplasia acid mucin	0.342	0.006**	0.740^++^	0.000**
Goblet cell metaplasia acid-sulfated mucin	0.330	0.008**	Undefined	Undefined
Proliferation (BrdU index) total versus				
Goblet cell metaplasia	0.392	0.001**	0.022	0.840
Goblet cell metaplasia mixed mucin	0.392	0.001**	0.098	0.365
Goblet cell metaplasia acid mucin	0.263	0.033*	−0.085	0.432
Goblet cell metaplasia acid-sulfated mucin	0.392	0.001**		
Goblet cell metaplasia total versus				
Goblet cell metaplasia mixed mucin	0.567^++^	0.008	0.467++	0.001**
Goblet cell metaplasia acid mucin	0.261	0.008	0.898+++	0.000
Goblet cell metaplasia mix mucin versus				
Goblet cell metaplasia acid mucin	0.331	0.008	0.194	0.072
Goblet cell metaplasia acid-sulfated mucin	0.331	0.008		

+++=Very strong (coefficient correlation >0.8), ++=Strong (coefficient correlation 0.6–0.8), +=Moderate (coefficient correlation 0.4–0.6),

** =p-value (p) <0.05,

* =p-value (p) <0.0, BrdU=Bromodeoxyuridine

BrdU-positive staining appeared by day 2 p.i. in hamsters and by day 1 p.i. in mice (Figure 1iii G). Both species showed abundant BrdU-positive cells on days 7 (Figure 1iii C and I) and 14 p.i. (Figure 1iii D, J). In hamsters, proliferation persisted into the chronic phase (Figure 1iii E and F), whereas in mice, BrdU reactivity declined to undetectable levels by day 56 p.i. (Figure 1iii L). BrdU-immunoreactive cells were absent in non-infected animals (data not shown).

The BrdU index reflects bile duct proliferation and is correlated with hyperplasia. Significant correlations were found between proliferation and hyperplasia, with coefficients of 0.748 in hamsters and 0.413 in mice (Table 3), both significant at the 0.000 level (two-tailed).

### GCM, mucin types, and their production

#### GCM

GCM is a key histopathological change in the bile duct mucosa of OV-infected hosts, where cholangiocytes transform into mucus-producing glands. This transformation was predominantly observed in first-order bile ducts and was absent in normal, non-infected biliary epithelial cells. This study detected goblet cells and their mucin production using histochemical staining. A histochemical study using the combination of AB-PAS staining distinguished between acid and neutral mucins, thereby coloring them blue and magenta, respectively. A mixture of both mucin types appeared purple. Sulfated acid mucin in goblet cells was detected using HID staining, which produced a dark brown-black coloration.

#### Mucin types

**Figure 2:**
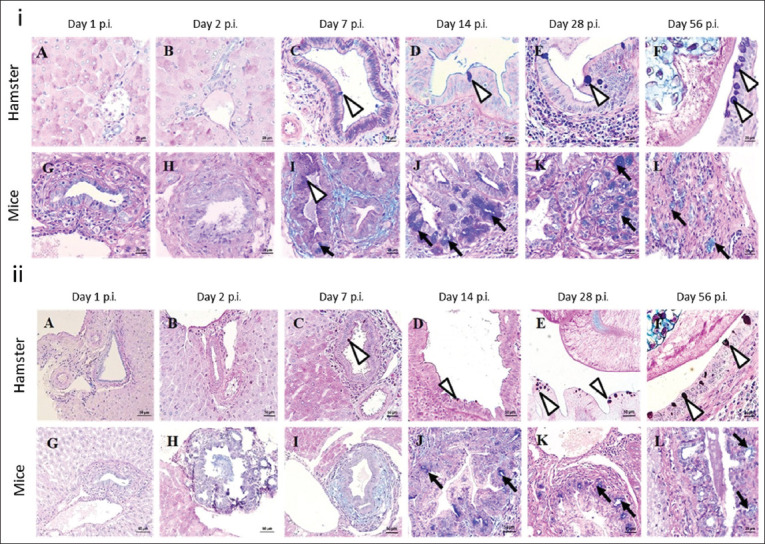
Kinetic changes in goblet cell metaplasia of the biliary tract of hamsters and mice at different times *of Opisthorchis viverrini* infection. (i) Various location, (ii) mucin types in the IHBD, and (iii) mucin type production (mucin index). GCM=Goblet cell metaplasia, IHBD=Intrahepatic bile duct, CBD=Common bile duct, GB=Gall bladder. *Indicated p < 0.05, **indicated p < 0.01, ***Indicated p = 0.000.

GCM and mucin types in the intrahepatic and extrahepatic biliary systems of OV-infected hamsters and mice were compared using the criteria presented in Table 3. GCM was absent in the early phase of infection (Figure 2i A, B, G, H). Purple-stained goblet cells containing mixed mucin (neutral and acid) (arrowheads) were predominant in hamster bile ducts from days 7 to 56 p.i. (Figure 2i C-F) but only on day 7 in mice (Figure 2i I). Mice displayed dramatic GCM with acid mucin overproduction (arrow) from days 14 p.i. to 28 p.i. and diminished by day 56 p.i. (Figure 2i J-L). The intrahepatic bile ducts, common bile ducts, and gallbladder were also examined in detail.

Metaplasia location varied according to the infection timeline: mice had pronounced lesions in the intrahepatic and common bile ducts during the late acute and sub-acute phases, peaking on days 2, 7, and 14 p.i., with day 7 prominence in the common bile duct. In contrast, hamsters showed significant GCM during the chronic phase. No distinct GCM pattern was observed in either species. In the intrahepatic region, acidic mucin in mice differed significantly from mixed, acidic, and acid-sulfated mucin in hamsters, as well as from mixed and acid-sulfated mucin in mice (p < 0.05). In the extrahepatic bile duct, hamster acid mucin differed significantly from mouse acid mucin and acid-sulfated mucin in the intrahepatic region (p < 0.05). In addition, acid-sulfated mucin in the CBD of hamsters was significantly different from mouse acid-sulfated mucin and intrahepatic acid-sulfated mucin (p < 0.05).

#### Histochemical analysis of GCM

Histochemical analysis revealed acid, neutral, mixed, sulfated, and non-sulfated acid mucins in OV-infected hamsters and mice at different infection stages (Figure 2). GCM scores were graded based on the criteria in Table 3. No mucin-containing goblet cells were detected in non-infected hamsters and mice. Mice exhibited more pronounced GCM and mucin production than hamsters. Mixed mucins predominated in the hamster bile ducts from days 7 to 56 p.i. (Figure 2C-F), whereas acid mucins were dominant in mice, particularly during the early infection phase (Figure 2H and I).

Mice showed a dramatic increase in mucin production from days 7 to 14 p.i., declining after day 14 as OV was presumably expelled. In contrast, hamsters showed a steady increase in mucin production, reaching a plateau that persisted until day 56 p.i. In the early phase of infection, metaplastic cholangiocytes in mice predominantly produced mixed mucins, shifting to acid mucins after day 14. In contrast, hamsters primarily produced mixed mucins later in the chronic phase, coinciding with declining mucin production in mice (Figure 2).

Regarding the sulfated and non-sulfated types, GCM in hamsters involved sulfated acid mucins and was stained dark brown with HID (Figure 2, arrowhead). In mice, non-sulfated mucins predominated, not staining with HID (Figure 2ii C and F, arrow). Hamsters showed an increase in GCM, consisting of mixed mucins, including the sulfated acid type, whereas mice mainly showed non-sulfated acid mucin (Figure 2ii J-I, arrows).

#### Mucin production

**Figure 3:**
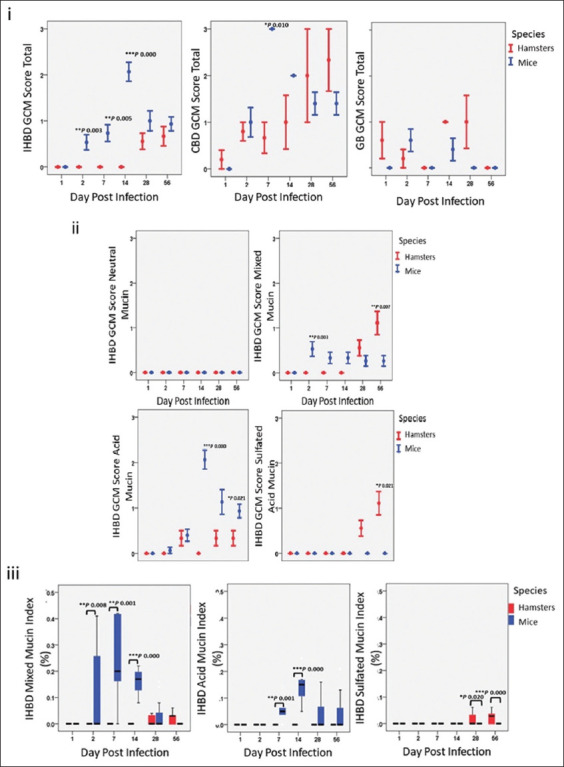
The differences between animal models are demonstrated. (i) Graphs indicating goblet cell metaplasia location, (ii) mucin-type, and (iii) mucin-type production.

Mucin production in the intrahepatic bile ducts of OV-infected hamsters and mice was quantitatively assessed through morphometric analysis, focusing on mixed, acid, and sulfated acid mucin types. The comparative mucin levels over the infection timeline are shown in Figure 3. No mucin-containing goblet cells were detected in uninfected animals. Infected mice exhibited significantly higher production of mixed mucins than hamsters as early as day 2 p.i. (p = 0.008). Mixed mucin levels in mice increased sharply by day 7 (p = 0.001), peaking on day 14 (p < 0.001), with an index value of approximately 0.2%. The mucin indices in mice declined from day 28 to day 56 p.i., whereas the hamster’s mucin production increased during this period. The mice showed pronounced overexpression of acid mucins during the sub-acute phase on days 7 and 14 p.i. (p = 0.001 and p < 0.001, respectively). In contrast, hamsters exhibited significant production of sulfated acid mucins during the chronic phase, with marked differences on day 28 (p = 0.001) and day 56 (p < 0.001). These findings revealed distinct mucin production patterns in the two models. Mice predominantly overexpressed non-sulfated acid mucins, whereas hamsters mainly produced sulfated acid mucins, especially during the chronic phase (Figure 3). The increased production of non-sulfated acid mucins in mice was most notable between days 14 and 28 p.i. (Figure 3). Importantly, no neutral mucin was detected in the intrahepatic bile ducts of the two species.

### Statistical analysis

Statistical analysis of histopathological features in the two animal models revealed significantly higher proliferation activity in mice during the early phase and in hamsters during the late phase (Table 4). In mice, cell proliferation activity was notably higher from day 1 p.i. (BrdU index p = 0.000), day 2 (Hyperplasia p = 0.002, BrdU index p = 0.000, GCM total p = 0.028), day 7 (GCM total p = 0.014), and day 14 p.i. (GCM total p = 0.000), with prominent acid mucin (GCM acid mucin p = 0.000). Alternatively, the biliary proliferation activity in the hamster model was observed in the late phase, beginning on day 28 p.i. (Hyperplasia p = 0.014, BrdU index p = 0.000) and continuing until day 56 p.i. (BrdU index p = 0.000). Regarding mucin types, hamsters showed significantly higher GCM acid-sulfated mucin on day 28 p.i. (p = 0.002) and day 56 p.i. (p = 0.001). Spearman’s correlation analysis revealed a very strong correlation between hyperplasia and the BrdU index in hamsters (r_s_ = 0.825, p = 0.000), a strong correlation between hyperplasia and GCM in mice (strong r_s_ = 0.799, p = 0.000), and a strong correlation between hyperplasia and GCM acid mucin in mice (strong r_s_ = 0.740, p = 0.000). Furthermore, a very strong correlation of GCM versus GCM acid mucin in mice (r_s_ = 0.898, p = 0.000) and a strong correlation between GCM and mixed mucin (r_s_ = 0.467, p = 0.001) in both mice and hamsters (r_s_ = 0.567, p = 0.000) (Table 3).

**Table 4:** Statistical analysis of histopathological feature in the intrahepatic bile duct of infected animals on different days of infection.

Biliary lesions and types of mucin	Day 1			Day 2			Day 7			Day 14			Day 28			Day 56		
					
	H	M	p-value	H	M	p-value	H	M	p-value	H	M	p-value	H	M	p-value	H	M	p-value
Hyperplasia	16.00	15.00	0.317	10.87	19.43	0.002**	14.22	10.57	0.163	9.06	14.57	0.031*	13.33	11.14	0.014*	12.17	12.70	0.845
Proliferation (BrdU index) total	11.00	20.00	0.000**	10.30	20.04	0.000**	11.50	12.32	0.628	12.50	12.50	1.000	18.50	7.82	0.000**	20.00	8.00	0.000**
Goblet cell metaplasia (GCM) total	15.50	15.50	1.000	13.00	17.14	0.028*	8.50	14.25	0.014*	5.00	17.00	0.000**	10.00	13.29	0.226	9.00	11.80	0.280
GCM neutral mucin	15.50	15.50	1.000	15.50	15.50	1.000	12.00	12.00	1.000	12.50	12.50	1.000	12.00	12.00	1.000	11.00	11.00	1.000
GCM mix mucin	15.50	15.50	1.000	13.00	17.14	0.028*	10.00	13.29	0.084	10.00	14.00	0.057	13.89	10.79	0.206	14.00	9.80	0.096
GCM acid mucin	15.50	15.50	1.000	15.50	15.50	1.000	11.83	12.11	0.909	5.50	16.70	0.000**	9.00	13.93	0.067	5.00	13.40	0.002**
GCM acid- sulfated mucin	15.50	15.50	1.000	15.50	15.50	1.000	11.50	11.50	1.000	11.50	11.50	1.000	15.89	9.50	0.002**	16.00	9.00	0.001**

GCM=Goblet cell metaplasia, BrdU=Bromodeoxyuridine, H=Hamsters, M=Mice. The significant values between animals at each time point are shown as

* p < 0.05 and

** p<0.01 of Mann–Whitney U-test

## DISCUSSION

In the pathological sequelae of biliary epithelium in OV infection, the proliferation lesion begins with hyperplasia before developing into the next pathological changes, such as dysplasia, cholangiofibrosis, or even bile duct cancer, and CCA [12]. Our study revealed significantly higher proliferation activity (hyperplasia, BrdU index, GCM) in the early stage of infection in the mouse model than in the hamster model. In mice, proliferation began immediately after infection, with significant increases observed from day 1 p.i. and continuing through day 14, particularly in association with increased acid mucin production. In contrast, biliary proliferation in hamsters was delayed, starting on day 28 p.i. and continuing until day 56. Hamsters also exhibited significantly higher levels of acid-sulfated mucin on day 28 and persisted until day 56 p.i. In addition, hamsters exhibited higher levels of acid-sulfated mucins on days 28 and 56 p.i.

BrdU, a thymidine analog, integrates into DNA during replication, making it a sensitive marker of cell proliferation, particularly in the S-phase. This method is widely used to quantify proliferating cells [36]. This study revealed that biliary hyperplasia was strongly correlated with the proliferation index (BrdU) in the hamster model (r_s_ = 0.825, p = 0.000). In the mouse model, a strong correlation between hyperplasia and GCM (r_s_ = 0.799, p = 0.000) is noted, particularly in relation to acid mucin-type in mice (r_s_ = 0.740, p = 0.000).

GCM, a notable histopathological change in OV-infected hosts, involves the transformation of cholangiocytes into mucin-secreting goblet cells. This metaplastic change, which resembles intestinal metaplasia, reflects the physiological similarities between the intrahepatic biliary and gastrointestinal tracts [37–39]. Our findings suggest that cholangiocytes respond to the fluke and adopt an intestinal profile during OV infection, transforming into goblet cells that produce increased mucin levels. In this experiment, GCM was more remarkable in mice than in hamsters within 28 days of infection. This process potentially contributes to worm expulsion through alterations in mucin composition. This adaptive response may represent a host defense mechanism characterized by increased numbers of goblet cells and mucin modifications. This adaptive response may represent a host defense mechanism characterized by increased numbers of goblet cells and mucin modifications. The host then displayed a protective mechanism by increasing the number of goblet cells and modifying the mucins. In addition to quantitative changes, a qualitative alteration of mucin, particularly acid mucin, was significant in helminth parasitism [14, 16, 40]. Furthermore, microbiodata play an important role in the development of healthy mucus for mucosal protection [41].

Mucins are large O-glycoproteins with high carbohydrate content and significant diversity in both their apoprotein and oligosaccharide components. They play a crucial role in maintaining cellular functions, especially on epithelial surfaces. Special stains or histochemical techniques can detect mucin alterations based on carbohydrate composition. Histochemical staining confirmed distinct mucin changes in the two models. AB staining effectively differentiated between acidic and neutral mucins. Neutral mucins, such as glucose, mannose, and galactose, lack charge, whereas acidic mucins contain negatively charged groups (carboxylate or sulfonate). These anionic groups bind to the cationic dyes. AB, with copper atoms and positively charged isothiouronium groups, selectively stains acidic mucins, whereas neutral mucins remain unstained. Our results confirmed AB’s ability to distinguish acidic from neutral mucins. Acidic mucins also bind to ferric cations, staining black with iron. Neutral mucins are stained magenta by PAS due to aldehyde group reactivity. A combination of AB and PAS stain tissues with both acidic and neutral mucins purple, reflecting their charged nature [42, 43]. Mice exhibited predominantly non-sulfated acid mucins, whereas hamsters exhibited mixed mucins, including the sulfated acid type. These differences highlight the divergent roles of mucins in the host-parasite interaction, with non-sulfated acid mucins in mice potentially contributing to worm expulsion and sulfated mucins in hamsters facilitating worm survival.

Previous studies by Hasnain *et al*. [14], Cortés *et al*. [44], and Cortes *et al*. [45] have linked mucin alterations with intestinal worm expulsion. The mucins had changed to sialylated or sulfated after infection [14, 16, 46]. However, our results suggest that sulfated acid mucins are not effective against OV, as they were primarily found in the hamster model, in which worms persisted. The results suggest that worms use mucins as a nutritional source essential for their survival. In contrast, the non-sulfated mucins in mice, which were significantly elevated on days 14 and 28 p.i., likely play a protective role. Based on these results, hosts may use different mucins as part of the mucosal innate protective mechanism [43]. Sulfated mucin found increasing in the bile duct of the hamster could not eliminate OV since this mucinous type was digestible by Ov-M60-like-1 [47]. Our data align with previous studies on the role of GCM and its mucin product in biliary epithelial cells with intestinal profile [14, 18, 19, 48-51]. Mucin overproduction in susceptible hosts, such as hamsters infected with *Echinostoma caproni*, supports worm survival in the host intestine [18, 50, 51]. The study in the susceptible mice breed for toxic agents (ICR) revealed that goblet cells in *E*. *caproni*-infected ICR mice intestinal villi are thoroughly lost with parasite retention [49]. Intriguingly, recent evidence on rat and mouse susceptibility differs. Rats as low-compatible hosts display mucin downregulation correlated with early worm removal from the intestine lumen. Then, a susceptible host mouse upregulated mucin levels so the worms were not expelled [45]. The upregulation of a pro-inflammatory cytokine, interferon-gamma (IFN-Y), and an enzyme catalyzing the formation of nitric oxide (iNOS) under the Th1 immune response probably facilitated the existence of *E caproni* in CD1 mice. In low-compatible rats, the Th2/Th17 types exhibit immune responses with a prominent level of interleukin (IL)-13 discharged from adult worms [52–55]. Furthermore, increasing the degree of GCM in hamsters in the late phase of infection was not followed by OV elimination, but it may facilitate worm establishment. Mucin alteration is considered under the Th2-type immune response [56–59] by the IL-4 and IL-13 consequence to its penetrability and stickiness might trap the worm and inhibit its feeding ability [44, 45]. In agreement with previous findings, GCM and mucin overexpression in mice may be associated with IL-13 upregulation, which changes the viscosity of the mucus and immobilizes the fluke, as dead OVs were observed on day 28 p.i. The above-mentioned profile was crucial in the non-susceptible rodent model of the worm expulsion mechanism [18, 19].

## CONCLUSION

This study highlights the distinct roles of GCM and mucin production in modulating host defense and parasite survival during OV infection in susceptible (hamsters) and non-susceptible (mice) hosts. Mice exhibited early and robust biliary responses, characterized by significant hyperplasia, GCM, and acid mucin overproduction, facilitating parasite expulsion by day 28 p.i. In contrast, hamsters demonstrated delayed responses with predominant sulfated mucin production, supporting parasite persistence and chronic infection. These findings underscore the dual role of mucins as both a defense mechanism and a potential facilitator of parasite survival, reflecting species-specific host-pathogen interactions.

The study’s strength lies in its comprehensive use of histochemical, immunohistochemical, and statistical methods to characterize biliary changes over time, offering new insights into the dynamics of mucosal immunity in OV infection. The comparative approach using two distinct rodent models enhances our understanding of susceptibility and resistance mechanisms, providing a robust framework for future studies.

However, the study has limitations. It focuses on rodent models, which, while informative, may not fully replicate the complexity of human responses to OV infection. In addition, the specific molecular mechanisms driving mucin production and its regulatory pathways were not explored in depth, leaving gaps in understanding the interplay between mucosal immunity and parasite biology.

Future research should aim to investigate the molecular pathways governing mucin alterations and GCM, including the role of cytokines and signaling cascades. Exploring microbiota contributions to mucosal changes and validating findings in human populations would strengthen translational relevance. Furthermore, the potential of mucin profiles as biomarkers for susceptibility, disease progression, or therapeutic targets warrants further investigation.

In conclusion, this study provides valuable insights into the histopathological changes associated with OV infection, offering a foundation for developing targeted interventions to mitigate the burden of this parasitic disease.

## AUTHORS’ CONTRIBUTIONS

ST, PT, and SS: Conceptualization. ST, WDW, and PS: Methodology. WS and ST: Validation. PeS and ST: Formal analysis. WDW, TT, and ST: Investigation. WDW and ST: Writing – original draft preparation. ST, WS, SS, and PS: Writing – review and editing. PT and ST: Supervision and editing. All authors have read and approved the final manuscript.
